# Change in cardiorespiratory fitness on self-rated health: prospective cohort study in 98 718 Swedish adults

**DOI:** 10.1177/14034948211047140

**Published:** 2021-10-19

**Authors:** Tobias Holmlund, Victoria Blom, Erik Hemmingsson, Björn Ekblom, Gunnar Andersson, Peter Wallin, Elin Ekblom-Bak

**Affiliations:** 1 Department of Physical Activity and Health, The Swedish School of Sport and Health Sciences, Sweden; 2 Research Department, HPI Health Profile Institute, Sweden

**Keywords:** Public health, self-reported health, sleeping problems, stress, pain, exercise, cardiorespiratory fitness

## Abstract

*Aim:* To study how change in cardiorespiratory fitness over time is associated with the development of poor self-rated health in healthy Swedish adults, and whether this association varies with sex, age, body mass index and cardiorespiratory fitness at baseline. A secondary aim was to study the influence of other predictors of self-rated health. *Methods:* A total of 98,718 participants (45% women, mean age 42.2 years) with two assessments from occupational health service screenings between 1988 and 2019 (mean duration 4.3 years), with good self-rated health at baseline were included. Cardiorespiratory fitness was assessed as estimated maximal oxygen consumption using submaximal cycle testing. Change in cardiorespiratory fitness was expressed as percentage annual change. Poor self-rated health at follow-up was defined as percieving self-rated health as ‘poor’ or ‘very poor’. *Results:* A large decrease in cardiorespiratory fitness (⩾−3%) was associated with a 34% higher risk of poor self-rated health compared to maintainers (−1 to +1%) after multi-adjustment including change in body mass index, back/neck pain, stress, exercise habits and sleep quality or sleep problems. The associations for decreasers were stronger with longer follow-up time (>10 years). Preserving, or changing to, risk level for body mass index, back/neck pain, stress, exercise and sleep quality/problems were associated with a higher risk of poor self-rated health. ***Conclusions:* Preserving or increasing cardiorespiratory fitness is associated with a lower risk of poor self-rated health, independently of change in other health-related variables, which may act as a protection against future poor self-rated health. This is of high clinical value, and strategies for maintaining or improving cardiorespiratory fitness have the potential to influence both disease and mortality.**

## Background

Self-rated health (SRH), i.e. the subjective perception of overall current health [1, 2], predicts several common future chronic diseases and mortality, independent of conventional risk factors such as blood pressure, diet, exercise, smoking, educational level and blood glucose [1, 3]. Social and demographic factors, such as age, gender, income, education level and social capital [3, 4] appear to play significant roles in determining SRH [1]. Moreover, depression, anxiety and low back pain are main contributors to the overall disease burden in the Nordic countries, which have an adverse influence on somatic diseases and mortality [5].

Cardiorespiratory fitness is an important component of overall physical fitness, which is mainly determined by moderate to vigorous intensity physical activity. Cardiorespiratory fitness has previously been found to be a strong independent predictor of cardiovascular disease and mortality in healthy men and women. Moreover, participants with high cardiorespiratory fitness have been reported as having higher SRH compared to participants with low cardiorespiratory fitness, after adjustment of conventional risk factors [6]. A combination of good/excellent SRH and moderate/high cardiorespiratory fitness has been associated with a 58% lower mortality risk compared to poor/fair SRH and low cardiorespiratory fitness [7]. While such associations are noteworthy, there is little information on how changes in cardiorespiratory fitness in adulthood can influence changes in SRH. Whether or not this is independent of change in other relevant lifestyle factors would add important knowledge about a potential direct association between cardiorespiratory fitness and SRH.

The aim of this study was to explore how changes in cardiorespiratory fitness were associated with the development of poor SRH in a large sample of adult men and women. A secondary aim was to study the influence of other predictors of SRH, including body mass index (BMI), back/neck pain, stress, exercise and sleep, in relation to cardiorespiratory fitness.

## Methods

### Recruitment and data collection

The present study is based on data from the health profile assessment (HPA) database managed by the HPI Health Profile Institute (HPI, Stockholm, Sweden). The HPA is an interdisciplinary method of assessing current lifestyle, health experiences and anthropometrics, and has been carried out through Swedish occupational health services since the middle of the 1970s. A HPA contains a questionnaire about lifestyle and health experiences, measurements of anthropometrics and blood pressure, an estimation of maximum oxygen consumption (VO_2max_) using a submaximal cycle test, and a brief dialogue with a HPA ‘coach’ to promote health and wellbeing. Participation in a HPA is optional and offered free to all employees working for an organisation connected to occupational or other health services in Sweden. All data from the HPA is registered and stored in a central database. The HPI is responsible for standardisation of the methods and the education of HPA coaches. For the present analyses, we included all participants who completed at least two HPAs since the establishment of the central database in April 1988 until November 2019, and had valid measurements of SRH and cardiorespiratory fitness on both occasions. For participants with more than two HPAs, the first and the latest valid HPA was used. Exclusion criteria were rating of SRH as ‘poor’ or ‘very poor’ at the first assessment. In total 108,476 participants fulfilled the criteria. To minimise influence of uncertainties in the data collection, we excluded those who had an annual increase/decrease in cardiorespiratory fitness of more than 50% (*n*=142), were younger than 18 years of age (*n*=9), had less than 90 days between tests (*n*=1531) or had missing data for stratification or confounding variables (*n*=8076). The original study adhered to the Declaration of Helsinki and was approved by the Stockholm ethics review board (Dnr 2015/1864-31/2 and 2016/9-32).

### Primary outcome

*SRH was assessed by using a single question outlined as follows ‘I perceive my overall general health to be. . .’ with five response alternatives including ‘very poor’, ‘poor’, ‘neither bad nor good’, ‘good’ or ‘very good’. The alternatives of replies were further dichotomised into ‘poor SRH’ (‘very poor’ or ‘poor’) and ‘not poor SRH’ (‘neither good nor bad general health’, ‘good general health’, and ‘very good general health’). A single item measure of general health has been shown to be as good as a multi-item measure to identify participants with an increased risk of mortality and hospitalisation [8].*

### Assessment of cardiorespiratory fitness

Cardiorespiratory fitness was assessed as estimated VO_2max_ using the standardised Åstrand submaximal cycle test [9], and expressed in relative (mL/min/kg) terms. To minimise well-known errors with submaximal testing, participants were requested to refrain from vigorous activity the day before the test, consuming a heavy meal 3 hours, smoking or using snuff one hour before the test, and avoiding stress. We have previously shown small and non-significant mean differences on a group level (–0.07 L/min; 95% confidence interval (CI) –0.21 to 0.06) between estimated VO_2max_ using the Åstrand test and directly measured VO_2max_ during treadmill running, with an absolute error and coefficient of variance (CV) similar to other submaximal tests (Standard error of estimate (SEE) 0.48 L/min; CV 18.1%) [10]. Cardiorespiratory fitness was further categorised into low (<32mL/min/kg), average (32–49 mL/min/kg) and high (≥50 mL/min/kg).

### Lifestyle-related variables and covariates

Body weight was assessed to the nearest 0.5 kg using a calibrated scale, and body height to the nearest 0.5 cm using a wall-mounted stadiometer. BMI was subsequently calculated (kg/m^2^). Current back or neck pain, overall stress, exercise habits, current sleep quality or sleep problems and intake of pain, sleep or mood medication were self-reported through the following statements: back or neck pain, ‘I perceive pain. . .’ with the alternatives of reply ‘very often’, ‘often’, ‘sometimes’, ‘rarely’ or ‘never’; overall stress, ‘I perceive stress in life, both personally and at work. . .’ with the alternatives of reply ‘very often’, ‘often’, ‘sometimes’, ‘rarely’ or ‘never’; exercise habits, ‘I exercise/train. . .’ with the alternatives of reply ‘never’, ‘occasionally’, ‘1–2 times a week’, ‘3–5 times/week’ or ‘at least 6 times/week’; sleep quality, ‘I consider my sleep to be. . .’ with the alternatives of reply ‘very poor’, ‘poor’, ‘neither good nor bad’, ‘good’ or ‘very good’; and sleep problems, ‘I have trouble sleeping at night. . .’ with the alternatives of reply ‘very often’, ‘often’, ‘sometimes’, ‘rarely’ or ‘never’. Intake of mood medicine, medicine for pain relief or medicine for sleep aid were self-reported. Educational level was not directly assessed in the HPA, but derived by converting occupational codes from the Swedish standard classification of occupation (2012) into three education levels: primary school, secondary school, university/vocational, as well as a fourth group of participants without information about formal degree. A detailed description of the conversion method is presented elsewhere [11].

In the analyses, changes in the risk level of other lifestyle-related factors were considered. The definition of risk level was for: BMI, 30 kg/m^2^ or greater; back/neck pain, ‘very often or often’; stress, ‘very often or often’; exercise habits, ‘never or occasionally’; sleep quality, ‘very poor or poor’; and sleep problems, ‘very often or often’. Changes in the risk level of these variables were categorised into four groups: improved level (risk at baseline but not at follow-up), impaired level (no risk at baseline but at follow-up), maintained non-risk level (non-risk at baseline and non-risk at follow-up), and maintained risk level (risk at baseline and risk at follow-up). Change in the intake of medicine (mood, pain or sleep) is self-reported as yes or no and dichotomised as ‘0’ if changed from intake of any of the medication to no intake of any medication or ‘1’ changing from no intake of medication to intake of any of the medications. Intake of medication was further divided into four groups (1=no–no; 2=yes–no; 3=no–yes; 4=yes–yes).

### Statistical analysis

Change in cardiorespiratory fitness as a continuous variable was calculated as the difference in estimated VO_2max_ between the two assessments and described as a percentage of annual change from the first assessment. Cardiorespiratory fitness was further divided into five arbitrary groups based on the magnitude of annual change described as: large decreasers (⩾−3%), small decreasers (<−3% to ⩾−1%), maintainers (<−1%, to <1%), small increasers (⩾1% to <3%) and large increasers (⩾3%). This method of distributing cardiorespiratory fitness has previously been shown to have a high association with hypertension [12]. Differences in baseline characteristics between the five groups were tested by Kruskal–Wallis analyses (continuous variables) and the chi-square test of variance (categorical variables), adjusting for multiple comparisons. Categorical data are presented as percentages. Binary logistic regression was used to estimate odds ratio (OR) and 95% CI for poor SRH at second assessment, with adjustment for relevant covariates chosen due to their potential association with both cardiorespiratory fitness and SRH [13]. Model 1 was adjusted for sex, age and time between assessments; model 2 additionally adjusted for baseline cardiorespiratory fitness, medicine intake and educational level; and model 3 additionally adjusted for change in risk level of BMI, back/neck pain, stress, exercise habits and sleep quality or sleep problems between the two assessments. For analysis of change in other lifestyle-related variables, the five cardiorespiratory fitness groups were collapsed into three groups: decreasers (⩾−1%), maintainers (<−1%, to <1%) and increasers (⩾1%).

To test for interaction between the covariates and change in cardiorespiratory fitness (continuous), an interaction term was introduced in the regression analysis. Interactions were defined as *P*<0.05 for the interaction term. To study the interaction between cardiorespiratory fitness change and changes in the other lifestyle-related variables, the procedure described by Altman and Bland was used [14]. All analyses were performed using IBM SPSS (Statistical Package for the Social Science for Windows) version 25, 2017, SPSS Inc, Chicago, IL, USA.

## Results

**Figure 1.**
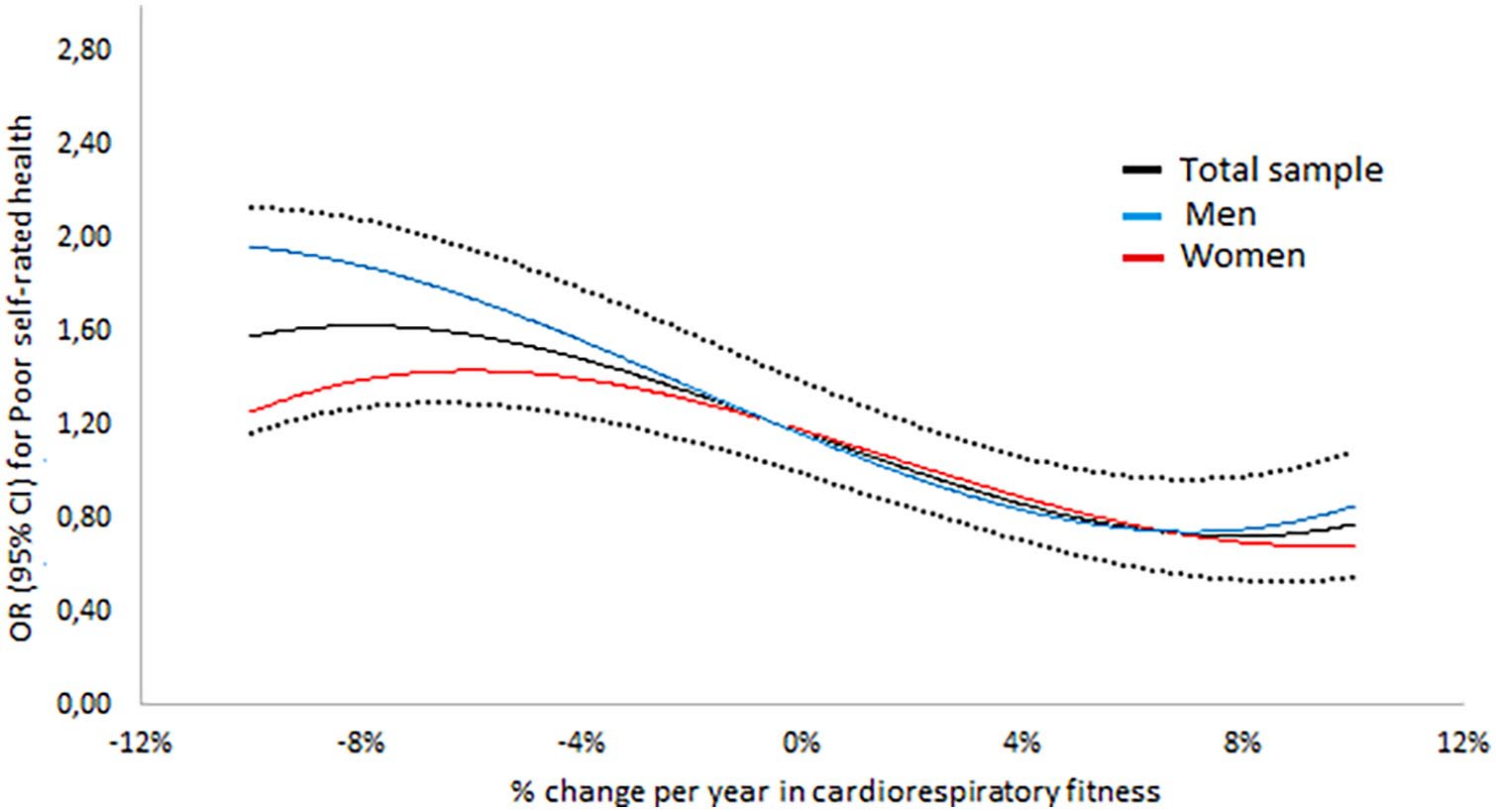
Odds ratios (ORs) (95% confidence interval (CI)) for continuous levels of yearly change (in %) in cardiorespiratory fitness for poor self-rated health (SRH) at second assessment.

A total of 98,718 participants (45% women, median age 42 years) were included. The mean duration between the two assessments was 4.3 years (standard deviation (SD) 3.6, ranging from 90 days to 28 years). Cardiorespiratory fitness (L/min) at baseline showed no difference between large and small decreasers but all other groups differed significantly (Table I). There was a significant difference between all groups for days between tests. Large increasers/decreasers had the shortest time between assessments compared to the other groups. ORs (95% CI) for poor SRH at second assessment in relation to continuous levels of change in cardiorespiratory fitness are presented in Figure 1.

**Table I.** Baseline characteristics of the study population in relation to five groups of annual change in cardiorespiratory fitness.

	Large decreasers	Small decreasers	Maintainers	Small increasers	Large increasers
	(⩾−3%)	(⩾−1 to <−3%)	(<−1. to <1%)	(⩾1.0 to <3%)	(⩾3%)
*N* (% women)	24 270 (46%)^a,b,c,d^	17 984 (40%)^b,c,d^	20 249 (44%)^d^	11 704 (43%)^d^	24 511 (48%)
Age (years)	43.3 (10.7)^a,b,c,d^	41.8 (9.9)^d^	41.9 (10.1)^c,d^	41.5 (9.9)^d^	41.5 (10.3)
Body weight (kg)	77.4 (14.7)	77.8 (14.5)^b,c,d^	77.2 (14.5)^c^	77.8 (14.6)^d^	77.3 (15.1)
Body height (cm)	173.8 (9.3)^a,b,c^	174.7 (9.1)^d^	174.3 (9.3)^d^	174.6 (9.1)^d^	173.6 (9.4)
BMI (kg/m^2^)	25.3 (22.8–27.6)^a,b^	24.9 (22.8–27.4)^d^	24.8 (22.7–27.3)^d^	24.9 (22.8–27.5)	25.0 (22.8–27.7)
Estimated VO_2max_ (L/min)	3.0 (0.8)^b,c,d^	3.0 (0.8)^b,c,d^	2.8 (0.7)^c,d^	2.7 (0.7)^d^	2.5 (0.7)
Days between baseline and follow-up	865 (401–1455)^a,b,c,d^	1970 (1112–3145)^b,c,d^	1955 (985–3294)^c,d^	1481 (879–2426)^d^	714 (365–1191)
Back/neck pain (very often/often)	15.8%^a,b,c^	14.1%^d^	14.2%^d^	14.5%^d^	15.7%
Overall stress (very often/often)	11.8%	11.8%	12.4%	11.8%	12.2%
Current exercise (never/occasionally)	29.4%^a,b,c^	32.2%^b,c,d^	34.4%	34.7%	35.2%
Sleep quality/problems (very often/often)	6.9%^a,b,c^	5.4%^d^	5.3%^d^	5.6%^d^	6.7%
Medicine mood/pain/sleep (very often/often)	6.9%^a,b,c^	5.0%^c,d^	5.1%^c,d^	5.8%^d^	6.7%

a Significantly different from small decreasers.

b Significantly different from maintainers.

c Significantly different from small increasers.

d Significantly different from large increasers.

Data shown as mean (standard deviation (SD)) or median (Q1–Q3).

BMI; body mass index.

Compared to maintainers (set as reference), ORs for poor SRH were significantly higher for large (OR 1.40, 1.25–1.57) and small (1.20, 1.07–1.34) decreasers. (Table II, model 1). Small and large increasers had similar ORs as maintainers. Adjustments including baseline cardiorespiratory fitness, change in medicine intake and educational level (model 2) and changes in BMI, back/neck pain, stress, exercise habits and sleep quality or sleep problems s between the two assessments (model 3) only slightly modified the associations. As there could have been a variation in SRH between participants included in the analyses (answer of reply varied between ‘neither bad nor good’, ‘good’ or ‘very good’), additional sensitivity analyses were performed including SRH at baseline, showing small alterations in the results (data not shown). There were significant interactions between cardiorespiratory fitness change (continued levels) and time between tests (*P*=0.001), sex (*P*=0.030), cardiorespiratory fitness at baseline (*P*=0.020) and change in BMI (*P*=0.049). No interactions were seen for cardiorespiratory fitness change and medicine change (*P*=0.243), educational level (*P*=0.098) and change in sleep quality or sleep problems (*P*=0.065), change in overall stress (*P*=0.200), change in back/neck pain (*P*=0.122) and change in exercise habits (*P*=0.568).

**Table II.** Odds ratios (95% confidence intervals) for poor self-rated health (SRH) at follow-up in relation to annual change in cardiorespiratory fitness and different follow-up time.

	Large decreasers (⩾−3%)	Small decreasers (⩾−1% to <−3%)	Maintainers (<−1% to <1%)	Small increasers (⩾1% to <3%)	Large increasers (⩾3%)	*r* ^2^
Model 1	1.40 (1.25–1.57)	1.20 (1.07–1.34)	1 (ref)	1.05 (0.92–1.20)	0.89 (0.79–1.01)	0.01
Model 2	1.53 (1.36–1.71)	1.29 (1.15–1.44)	1 (ref)	1.00 (0.87–1.14)	0.80 (0.71–0.91)	0.07
Model 3	1.34 (1.19–1.52)	1.20 (1.07–1.36)	1 (ref)	1.08 (0.93–1.24)	0.89 (0.78–1.02)	0.23
Stratified for time between the two assessments^*^	Large decreasers (⩾−3%)	Small decreasers (⩾− 1% to <−3%)	Maintainers (<−1 %to <1%)	Small increasers (⩾1% to <3%)	Large increasers (⩾3%)	
3 months to 1 yr (*n*=15,904)	1.24 (0.87–1.77)	1.38 (0.85–2.24)	1 (ref)	0.89 (0.53–1.50)	0.77 (0.54–1.09)	0.19
1 to 5 yrs (*n*=51,390)	1.16 (0.98–1.37)	0.93 (0.78–1.17)	1 (ref)	0.93 (0.75–1.16)	0.78 (0.65–0.94)	0.24
5 to 10 yrs (*n*=22,698)	1.39 (1.10–1.75)	1.20 (0.98–1.46)	1 (ref)	1.18 (0.93–1.51)	1.04 (0.77–1.41)	0.23
≥10 yrs (*n*=8,731)	2.28 (1.31–3.96)	1.61 (1.22–2.13)	1 (ref)	1.13 (0.74–1.72)	0.73 (0.30–1.77)	0.23

Model 1 adjusted for sex, age and time between assessments.

Model 2 additionally adjusted for baseline cardiorespiratory fitness, change in medicine intake and educational level.

* Model 3 additionally adjusted for change in risk level of body mass index, back/neck pain, stress, exercise habits, and sleep quality or sleep problems.

### In relation to follow-up time and subgroups

**Figure 2.**
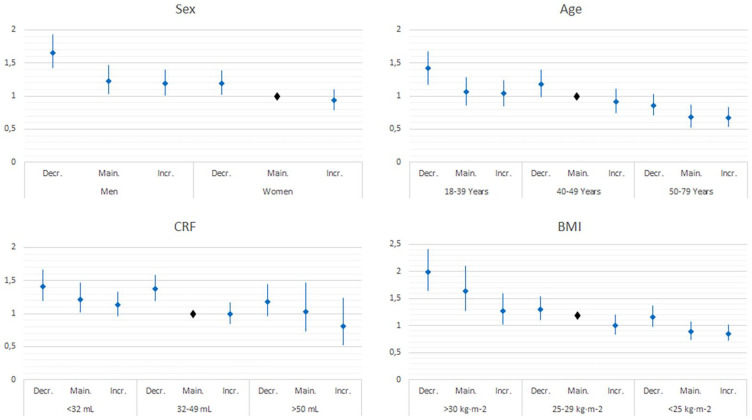
Odds ratios (ORs) (95% confidence interval (CI)) for poor self-rated health (SRH) in relation to annual change in cardiorespiratory fitness and subgroups of sex, age cardiorespiratory fitness and body mass index (BMI) at baseline. Adjusted for sex, age and time between assessments, baseline cardiorespiratory fitness, mood medicine intake, educational level, change in risk level of BMI, back/neck pain, stress, exercise habits and sleeping problems. Decr: decreasers; Main: maintainers; Incr: increasers; CRF: cardiorespiratory fitness.

Analysing the effect of time between the two assessments, large increasers in one to 5 years between assessments showed a significant lower association to poor SRH, on the contrary small and large decreasers in the longest time frame between assessments (≥10 years) showed significant associations with poor SRH at follow-up (Table II). Men, young age, low cardiorespiratory fitness and obesity were all associated with higher ORs for poor SRH at follow-up, compared to their counterparts (Figure 2). However, participants in a less conducive subgroup of sex, age, cardiorespiratory fitness and BMI, but with an increase in cardiorespiratory fitness between the two assessments, had a comparable association to poor SRH as a decreaser in a more conducive subgroup.

### In relation to change in other lifestyle-related variables

Changing from a non-risk to a risk level, or maintaining a risk level for BMI, back or neck pain, stress, exercise, sleep quality or sleep problems was associated with a higher OR for poor SRH at follow-up, compared to those who maintained or improved to a non-risk level (Table III, first and second left column). Maintaining or increasing cardiorespiratory fitness attenuated the OR for poor SRH both among risk and non-risk levels for BMI, back or neck pain, stress, exercise and sleep quality or sleep problems.

**Table III.** Odds ratios (95% confidence intervals) for poor self-rated health (SRH) in relation to change in body mass index (BMI), back/neck pain, stress, exercise habits and sleep quality or sleep problems between the two assessments (two left columns) and after cross-tabulation of change in CRF and change in the other lifestyle-related behaviours (three right columns).

Change in the other lifestyle-related behaviours	OR (95% CI) for poor SRH		Decreasers	Cardiorespiratory fitness change	Increasers
				Maintainers	
BMI					
Maintained non-obese (*n*=83,546)	1 (ref)	1 (ref)	1.25 (1.10–1.41)	1 (ref)	0.96 (0.84–1.09)
Changed to non-obese (*n*=1,953)	0.82 (0.61–1.11)				
Changed to obesity (*n*=3,877)	2.25 (1.95–2.60)	2.10 (1.90–2.31)	2.66 (2.29–3.09)	1.93 (1.58–2.36)	1.80 (1.50–2.17)
Maintained obesity (*n*=9,342)	1.99 (1.77–2.23)				
Back/neck pain					
Maintained sometimes/rarely/never (*n*=75,350)	1 (ref)	1 (ref)	1.24 (1.08–1.42)	1 (ref)	0.95 (0.82–1.10)
Changed to sometimes/rarely/never (*n*=7,669)	1.04 (0.90–1.21)				
Changed to often/very often (*n*=8,577)	2.78 (2.51–3.07)	2.60 (2.39–2.82)	3.28 (2.85–3.79)	2.49 (2.08–2.97)	2.39 (2.03–2.81)
Maintained often/very often (*n*=7,122)	2.43 (2.17–3.71)				
Stress					
Maintained sometimes/rarely/never (*n*=80,670)	1 (ref)	1 (ref)	1.37 (1.20–1.57)	1 (ref)	0.99 (0.86–1.15)
Changed to sometimes/rarely/never (*n*=7,725)	1.42 (1.23–1.63)				
Changed to often/very often (*n*=6,185)	5.63 (5.11–6.21)	5.25 (4.84–5.70)	6.69 (5.76–7.77)	6.02 (5.05–7.19)	5.41 (4.60–6.36)
Maintained often/very often (*n*=4,138)	5.40 (4.81–6.05)				
Exercise habits					
Maintained ⩾1 time/week (*n*=55,523)	1 (ref)	1 (ref)	1.23 (1.07–1.42)	1 (ref)	0.91 (0.78–1.06)
Changed to ⩾1 time/week (*n*=16,055)	1.20 (1.06–1.36)				
Changed to occasionally/never (*n*=10,638)	2.71 (2.43–3.01)	2.42 (2.23–2.61)	2.83 (2.46–3.26)	2.19 (1.85–2.61)	2.26 (1.92–2.66)
Maintained often/very often (*n*=16,502)	2.44 (2.21–2.70)				
Sleep quality or sleep problems					
Maintained good sleep (*n*=87,498)	1 (ref)	1 (ref)	1.31 (1.15–1.48)	1 (ref)	1.00 (0.87–1.15)
Changed to good sleep (*n*=3,538)	1.10 (0.92–1.33)				
Changed to poor sleep (*n*=5,219)	4.73 (4.30–5.22)	4.05 (3.71–4.41)	5.24 (4.52–6.08)	4.41 (3.66–5.31)	3.78 (3.19–4.49)
Maintained poor sleep (*n*=2,463)	2.85 (2.46–3.29)				

Adjusted for sex, age and time between assessments, baseline cardiorespiratory fitness, mood medicine intake, educational level, change in risk level of BMI, back/neck pain, stress, exercise habits and sleep quality or sleep problems.

## Discussion

### Main findings

The main findings in the present study were that both a large and a small annual decrease in cardiorespiratory fitness was associated with higher ORs for poor SRH at follow-up (34% and 20%, respectively), compared to maintainers. Participants who increased cardiorespiratory fitness between the assessments had similar ORs as maintainers. This was seen even after adjustment for baseline cardiorespiratory fitness, change in medicine intake, educational level and change in risk level of BMI, back/neck pain, stress, exercise habits and sleep quality or sleep problems. The associations with decreasers were strongest in those participants with the longest follow-up time between the two assessments (≥10 years).

Subgroup analyses showed that men, as well as younger and obese participants who decreased cardiorespiratory fitness had a higher association with poor SRH compared to women, older and normal weight participants who decreased their cardiorespiratory fitness. These results are in line with previous research in which sex (men), age and obesity have been shown to affect SRH [6, 15, 16]. Moreover, increasers in a less conducive subgroup of sex (men), age (younger), cardiorespiratory fitness (lower) and BMI (higher) had equal or attenuated risk associations for poor SRH compared to decreasers in a more conducive subgroup. For example, increasers among men and cardiorespiratory fitness between 32–49 mL and BMI of less than 25kg/m^2^ had a significantly lower risk for poor SRH compared to decreasers in the same group. Similarly, maintainers and increasers in the highest age group (50–79 years) had a significantly lower risk for poor SRH compared to maintainers/decreasers in the lower age groups (40–49 and 18–39 years).

The current study showed that those who maintained or changed to a risk level (BMI, back/neck pain, stress, exercise habits and sleep quality or sleep problems) had a higher association with poor SRH compared to those who maintained or changed to a non-risk level. Adding a decrease in cardiorespiratory fitness further increased the risk for poor SRH. On the contrary, an increase in cardiorespiratory fitness attenuated the risk for poor SRH. Interestingly, those who changed from a risk to a non-risk level for stress and exercise habits, independently of change in cardiorespiratory fitness, still had a higher association with poor SRH. In contrast, those who maintained a non-risk level between the two tests and increased cardiorespiratory fitness at follow-up attenuated the association with poor SRH compared to maintainers and decreasers.

### Previous research

We are not aware of any study that has explored the longitudinal association between change in cardiorespiratory fitness and SRH, and simultaneously adjusted for changes in other lifestyle-related variables [17, 18]. However, our result is in line with previous cross-sectional studies that examined the association between cardiorespiratory fitness and SRH. For example, higher levels of cardiorespiratory fitness were associated with an improved SRH [6]. Other longitudinal studies have found associations between cardiorespiratory fitness and mental health problems, such as stress-related exhaustion [17], depression [19] and sleep disturbance [20]. Previous studies have also shown that changes in other health-related variables such as BMI [16], back/neck pain [16], stress [21], exercise habits [22] and sleep problems add to the risk of poor SRH. For example, overweight/obesity and perceived pain have been have shown to be associated with lower SRH [16]. Moreover, high perceived stress has been associated with a 75% higher risk of poor SRH, compared to those who reported almost no stress [21]. Conversely, high cardiorespiratory fitness and physical activity is associated with a lower risk of poor SRH [6], better sleep [23] and less pain [24]. All things considered, increasing or maintaining cardiorespiratory fitness in adulthood, independently of change in other health-related variables, may act as a protection against future poor SRH, which is of high clinical value [25].

Possible mechanisms between an increase in cardiorespiratory fitness and change in SRH may be linked to structural changes that promote functions in brain regions linked to mental health [26, 27]. Moreover, exercise has the potential to reduce inflammation and increase the resilience to damage due to oxidative stress, both of which are implications for common health disorders [28, 29]. Rahman et al. [30] showed that increases in cardiorespiratory fitness were significantly associated with a reduction in depressive symptoms independent of the frequency and intensity of the exercise. This is in line with our results of the subgroup analyses, in which participants who increased their cardiorespiratory fitness in less conducive subgroups had equal or lower risk for poor SRH independent of time spent physically active. However, this seems to be more pronounced among younger participants and those with a higher BMI. Maintaining or increasing cardiorespiratory fitness has dual benefits – as a protection for mental health and improved cardiovascular health, which are important in future health strategies [31].

### Strengths and limitations

A limitation of the present study is the voluntary participation, which could affect the results because the cohort may be partly selective. Nonetheless, the wide range of both exposure and outcome from these types of associational studies is less influenced by selected populations. The use of a submaximal test to estimate VO_2max_ is another possible limitation, although measuring actual VO_2max_ would not be feasible in this large non-athletic population. The submaximal protocol used has been reported to yield valid and reliable estimations of actual compared to directly measured VO_2max_ [10]. Even though the data collection was not initially intended for research proposes, the standardisation and quality control is well suited for such analyses. The result might be influenced by inverse causality, people with poor SRH may refrain from physical activity so there is a bilateral association, a low level of physical activity predicts a low level of SRH, but also that low SRH might predict a low level of physical activity. The strengths of the study were the large population-based sample of both sexes, different ages, a large variation of and change in cardiorespiratory fitness between the assessments, as well as the assessment of change in other health-related variables. Another strength was the inclusion of two assessments of cardiorespiratory fitness; this limits the genetic contribution to cardiorespiratory fitness, which is determined on current levels of moderate to vigorous physical activity [32].

## Conclusions

This study demonstrates that maintaining or improving cardiorespiratory fitness between two assessments in adulthood is associated with lower risk of future poor SRH, regardless of sex, age, BMI status, cardiorespiratory fitness at baseline and changes in other health-related variables. Moreover, maintaining and improving cardiorespiratory fitness attenuated an amplified risk of poor SRH associated with other well-known risk factors such as BMI, stress and sleep quality or sleep problems. In the light of the ongoing decline in SRH in the Nordic countries and the influence on the overall burden of disease and mortality, strategies for maintaining or improving cardiorespiratory fitness have the potential to influence both disease and mortality. However, further research is warranted to conclude the causal relationship between longitudinal changes in cardiorespiratory fitness and SRH.
